# Protocol for high-quality single-cell RNA-seq from tissue sections with DRaqL

**DOI:** 10.1016/j.xpro.2024.103050

**Published:** 2024-05-02

**Authors:** Hiroki Ikeda, Shintaro Miyao, Nanami Yamada, Sumire Sugimoto, Fuminori Kimura, Kazuki Kurimoto

**Affiliations:** 1 Department of Embryology, School of Medicine, Nara Medical University, Kashihara, Nara 634-8521, Japan; 2 Department of Obstetrics and Gynecology, School of Medicine, Nara Medical University, Kashihara, Nara 634-8521, Japan

**Keywords:** Single Cell, Developmental biology, RNAseq

## Abstract

Single-cell RNA sequencing (scRNA-seq) combined with laser capture microdissection (LCM) offers a versatile framework for comprehensive transcriptomics from tissue sections. Here, we present a detailed protocol for DRaqL (direct RNA recovery and quenching for LCM) in combination with Smart-seq2 (DRaqL-Smart-seq2), which enables high-quality RNA sequencing for single cells obtained from alcohol-fixed murine ovarian sections. Additionally, we provide an optional procedure for scRNA-seq from formalin-fixed sections (DRaqL-Protease-Smart-seq2). We outline key steps for cell lysis, cDNA amplification, and sequencing library preparation.

For complete details on the use and execution of this protocol, please refer to Ikeda et al.^1^

## Highlights

• Steps for isolating single cells from fixed and stained ovarian sections
• Steps for cell lysis and quenching by sequentially using two different detergents
• Instructions for cDNA amplification directly from the cell lysates
• Instructions for library preparation of 3′-end and full-length RNA sequencing

## Abstract (editor highlights)

Publisher’s note: Undertaking any experimental protocol requires adherence to local institutional guidelines for laboratory safety and ethics.

## Graphical abstract

**Figure:**
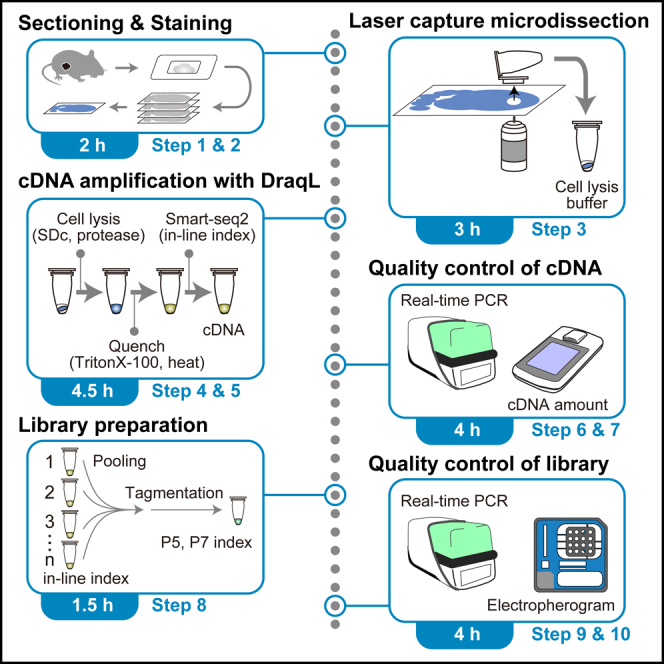

## Before you begin

**Figure 1:**
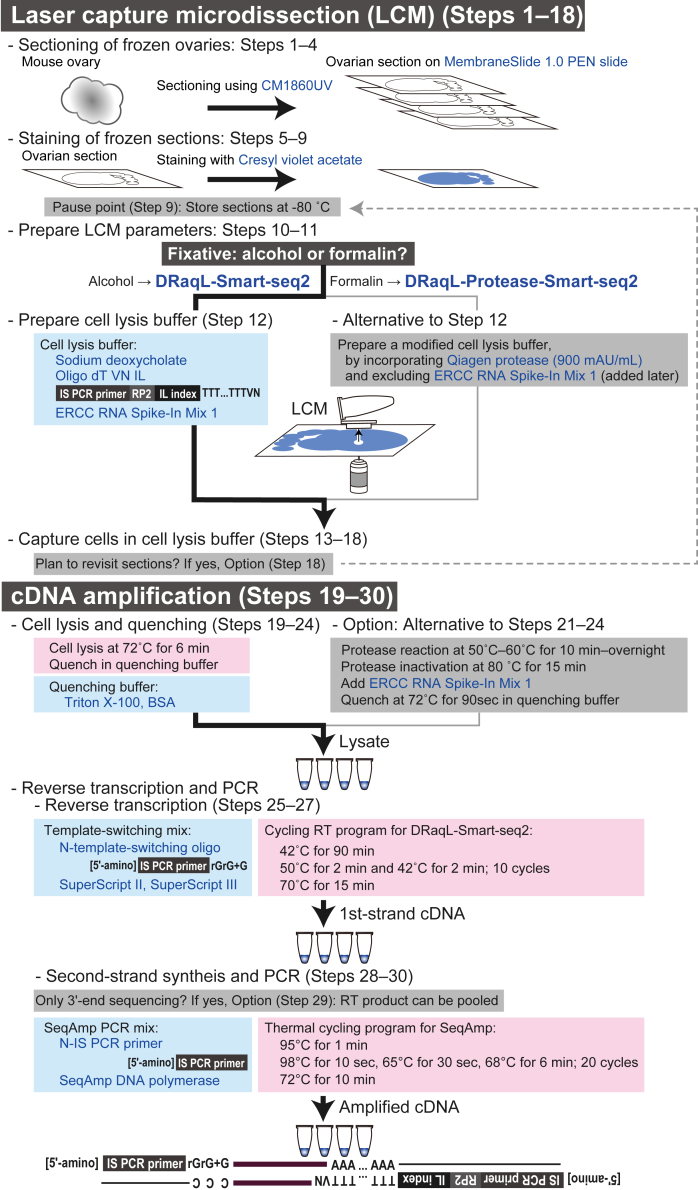
Outline of LCM and cDNA amplification

**Figure 2:**
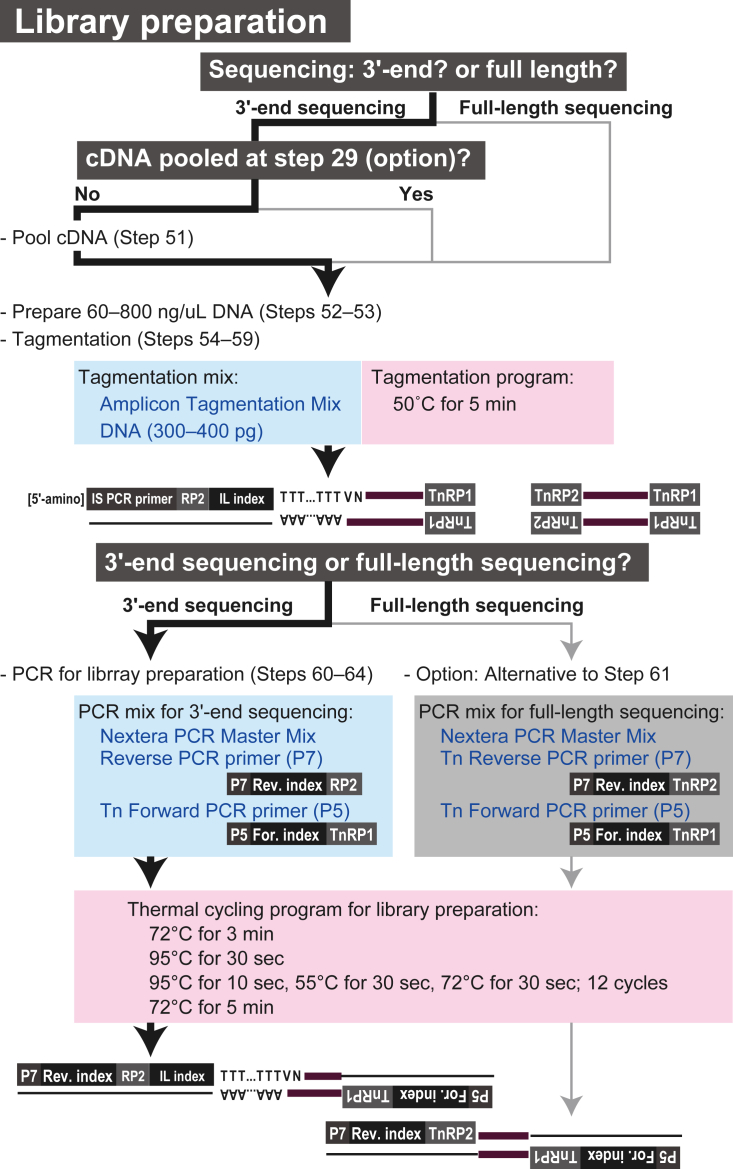
Outline of sequencing library preparation

DRaqL (direct RNA recovery and quenching for laser capture microdissection) allows high-quality RNA-sequencing (RNA-seq) for single cells isolated from fixed tissue sections by seamlessly combining efficient cell lysis and robust cDNA amplification. In DRaqL, cells within fixed sections are efficiently lysed using a denaturing detergent, sodium deoxycholate (SDc). The denaturing activity of SDc is subsequently quenched by adding an excess amount of a non-denaturing detergent, Triton X-100, which allows efficient enzymatic reactions for cDNA amplification. The following protocol outlines the specific steps of DRaqL combined with Smart-seq2 (DRaqL-Smart-seq2), optimized for alcohol-fixed, dye-stained sections. Cells can be isolated from sections using laser capture microdissection (LCM) or any other device. The sensitivity of single-cell RNA-seq from murine ovarian sections with DRaqL-Smart-seq2 is comparable to that of deep RNA-seq of freshly dissociated single cells.^1^ This protocol can be adapted to formalin-fixed sections by incorporating protease treatment (DRaql-Protease-Smart-seq2), as noted in Steps 12 and 21–24. The protocol overview is illustrated in Figures 1 and 2.

While this protocol focuses on Smart-seq2 for cDNA amplification, other cDNA amplification methods, namely SC3-seq^2^^,^^3^ and SMART-seq v4 3′DE Kit (Takara Bio, Z5040N), can also be combined with DRaqL.^1^

**Figure 3:**
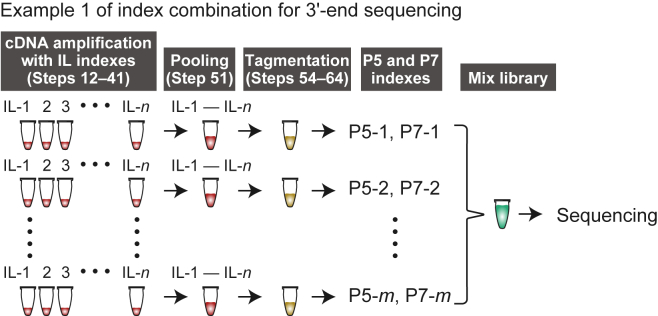
Example of index combinations An example of IL, P5, and P7 index combinations for 3′-end sequencing are shown. Note that the same IL index can be used to label cDNAs from different cells, as long as they are labeled with different P5 and dP7 indexes (see Notes at Step 12).

As outlined in Figure 3, the cDNA libraries are labeled with three different index sequences: in-line (IL) indexes incorporated in the reverse transcription primers (Oligo dT VN IL) (Step 12), and P5 and P7 indexes installed in PCR primers for sequencing libraries (Step 61). The IL index serves to distinguish 3′ ends of cDNAs from different cells, allowing the pooling of cDNAs after the first-strand synthesis (Step 29) or PCR amplification (Step 51). Subsequently, the P5 and P7 indexes allow pooling of the libraries for sequencing (Step 75).

Before starting with this protocol, it is crucial to prevent RNase contamination. For all RNA-related procedures in this protocol, we strongly recommend using RNase-free equipment including 10-μL, 200-μL, and 1000-μL tips, as wells as sampling tubes and PCR tubes. In addition, we recommend use of the Distilled water (dH_2_O) product specified in the key resources table throughout this protocol, or any other high-quality water product. Furthermore, we recommend conducting all procedures wearing disposable gloves and surgical masks in a clean laboratory environment. RNase Quiet or RNase ZAP may be employed to decontaminate the experimental workspace. When working with reaction tubes, use both hands to open and close the tube caps to prevent touching the inside, and avoid one-handed handling.

### Institutional permissions

Experiments involving mice, as described in this protocol, must be conducted in accordance with relevant institutional and national guidelines.

### Preparation of frozen ovaries

**Timing: 2 h**

Before beginning with this protocol, prepare frozen ovarian sections from an 8-week-old female mouse (see also Methods video S1).

1. Preparation of fresh ovaries.
  a. Prepare liquid nitrogen in a Styrofoam box or Dewar vessel.
  b. Humanely sacrifice an 8-week-old female mouse and excise the ovaries.
  c. Remove ovarian fat pads (adipose tissues adjacent to the ovaries) as much as possible using tweezers and surgical scissors.
  d. Wash the excised ovaries in 1×PBS.

***Note:*** Fat tissues are challenging to freeze at −20°C and can impede the sectioning of frozen tissues.

***Note:*** The inside of the Styrofoam box may be painted black to enhance visibility of the tissues.

2. Snap freezing of mouse ovaries.
  a. Chill embedding medium (10% PVA/1×PBS) or optimal cutting temperature (OCT) compound in a cryomold (Tissue-Tek Cryomold) on ice.
  b. Quickly freeze the ovaries by submerging them in liquid nitrogen.
  c. Embed the frozen ovaries in the chilled medium (Step a) and immediately return them to liquid nitrogen for freezing.
  d. Store the frozen ovaries embedded in the cryomold at −80°C.

Methods video S1. Method for snap freezing of mouse ovary, related to the preparation of frozen ovaries step in the before you begin section

## Key resources table

**Table:** 

REAGENT or RESOURCE	SOURCE	IDENTIFIER
**Chemicals, peptides, and recombinant proteins**		

Sodium deoxycholate	Nacalai Tesque	02889-72
Triton X-100	Nacalai Tesque	12967-32
Eosin Y stain solution (0.17% Eosin Y, 60% ethanol) (optional)	Muto Pure Chemicals	32051
Cresyl violet acetate	MP Biomedicals	150727
Distilled water (dH_2_O)	Thermo Fisher Scientific	15230-162
dNTP mixture (25 mM each)	Nippon Gene	312-07271
Dithiothreitol (DTT) (100 mM)	Thermo Fisher Scientific	Attached with Superscript
MgCl_2_ (1 M)	Merck	M1028-100ML
Betaine	Sigma	61962-50G
Ethanol	Wako	054-07225
Isopropanol	Wako	168-21675
Bovine serum albumin (BSA) (20 mg/mL)	Takara Bio	2320
Polyvinyl alcohol (PVA)	Sigma	P8136-250G
Optimal cutting temperature (OCT) compound (optional)	Sakura Finetek Japan	4583
1× Phosphate-buffered saline (PBS)	Nacalai Tesque	11480-35
Tris-HCl (pH 8.0) (1 M)	Nacalai Tesque	06938-44
Tween 20	Wako	166-21213
PhiX Control v3 DNA	Illumina	FC-110-3001
Sodium dodecyl sulfate (SDS) (alternative)	Wako	194-13402
Sodium N-lauroylsarcosinate (alternative)	Wako	121-05502
Sodium cholate (cholic acid sodium salt) (alternative)	Nacalai Tesque	06315-94
Polyoxyethylene (23) lauryl ether (Brij L23, Brij 35) (alternative)	Sigma	B4184-100ML

**Critical commercial assays**		

Superscript II	Thermo Fisher Scientific	18064014
Superscript III	Thermo Fisher Scientific	18080044
Recombinant RNase inhibitor (40 U/μL)	Takara Bio	2313A
RNaseOUT recombinant ribonuclease inhibitor (40 U/μL) (alternative)	Thermo Fisher Scientific	10777019
SeqAmp DNA polymerase	Takara Bio	638504
ERCC RNA Spike-In Mix 1	Thermo Fisher Scientific	4456740
PrimeScript II reverse transcriptase (alternative)	Takara Bio	2690A
QIAGEN protease (7.5 AU, lyophilized) (optional)	QIAGEN	#19155
AxyPrep MAG PCR clean-up kit	Corning	MAG-PCR-CL-50
Nextera XT DNA Library Prep Kit	Illumina	FC-131-1024
AMPure XP reagent (alternative)	Beckman Coulter	A63881
2× Power SYBR Green PCR master mix	Thermo Fisher Scientific	4368706
Qubit 1X dsDNA High Sensitivity (HS) and Broad Range (BR) Assay Kits	Invitrogen	Q33231
Agilent High Sensitivity DNA Kit	Agilent Technologies	5067-4626
KAPA Library Quantification Kits	Kapa Biosystems	KK4824
NextSeq 500/550 High-Output Kit v2.5 (75 Cycles)	Illumina	20024906
NextSeq 2000 P3 reagents (50 Cycles) (alternative)	Illumina	20046810

**Oligonucleotides**		

Oligo dT VN IL (100 μM): AAGCAGTGG TATCAACGCAGAGTACGTGTGCTCT TCCGATCT [*IL index*] TTTTTTTTTTTTT TTTTTTTTTTTTTTTTTVN (V: A/C/G. N: A/C/G/T) (HPLC grade): See Table S1 for full sequences including the IL index	Ikeda et al., 2023^1^	N/A
N-Oligo dT VN IL UMI (100 μM): [5AmMC6] AAGCAGTGGTATCAACGCAGAGTACGTGT GCTCTTCCGATCT [*IL index*] NNNNNNNNNV TTTTTTTTTTTTTTTTTTTTTTTTTTTTTTVN (HPLC grade) (alternative)	This study	N/A
N-template-switching oligo: [5AmMC6] AAG CAGTGGTATCAACGCAGAGTAC GCrGrG+G (r: ribonucleotide, +: LNA) (RNase-free HPLC grade)	Ikeda et al., 2023^1^	N/A
N-IS PCR primer: [5AmMC6] AAGCAGTG GTATCAACGCAGAGT (HPLC grade)	Ikeda et al., 2023^1^	N/A
Reverse PCR primer (P7 index): CAAGC AGAAGACGGCATACGAGAT [*P7-index*] GTGACTGGAGTTCAGACGTGTGCTCT TCCGATCT (HPLC grade): See Table S1 for sequences of the P7 index	Ikeda et al., 2023^1^	N/A
Tn Forward PCR primer (P5 index): AATGATA CGGCGACCACCGAGATCTACAC [*P5-index*] TCGTCGGCAGCGTC (HPLC grade): See Table S1 for sequences of the P5 index	Ikeda et al., 2023^1^	N/A
Tn Reverse PCR primer (P7 index): CA AGCAGAAGACGGCATACGAGAT [*P7-index*] GTCTCGTGGGCTCGG (HPLC grade) (alternative)	Ikeda et al., 2023^1^	N/A

**Software and algorithms**		

SMART-Seq_DE3_Demultiplexer	Takara Bio	https://www.takarabio.com/products/next-generation-sequencing/bioinformatics-tools/smart-seq-de3-demultiplexer
Umi_tools	Smith et al., 2017^4^	https://github.com/CGATOxford/UMI-tools
FASTQC	Andrews, 2010^5^	https://github.com/s-andrews/FastQC
Fastp	Chen et al., 2018^6^	https://github.com/OpenGene/fastp
HTSeq	Anders et al., 2015^7^	https://github.com/simon-anders/htseq
featureCounts	Liao et al., 2014^8^	https://subread.sourceforge.net/

**Other**		

Thermal cycler Mastercycler Nexus	Eppendorf	#6333 000.030
Laser microdissection system	Carl Zeiss	PALM MicroBeam 4
CFX Opus 384 Real-Time PCR system	Bio-Rad	#12011452J1
Qubit 4 fluorometer	Thermo Fisher Scientific	Q33226
Agilent 2100 Bioanalyzer	Agilent Technologies	2100 Bioanalyzer
Objective lens 20×	Carl Zeiss	Objective LD Plan-Neofluar 20x/0.4 Corr M27, 421350-9971-000
Objective lens 40×	Carl Zeiss	Objective LD Plan-Neofluar 40x/0.6 Corr M27, 421360-9970-000
Objective lens 63×	Carl Zeiss	Objective LD Plan-Neofluar 63x/0.75 Corr M27, 421381-9971-000
Objective lens 100× (optional)	Carl Zeiss	Plan-Apochromat 100x/1.4 Oil DIC, 420792-9901-000
Single flat-top 200-μL PCR tubes	Greiner Bio-One	#683201
Protein 1.5-mL LoBind tubes	Eppendorf	30108116
Protein 0.5-mL LoBind tubes	Eppendorf	30108094
DNA 0.5-mL LoBind tubes	Eppendorf	0030108035
0.6 mL SnapLock microtube Maxymum Recovery (alternative)	Corning	MCT-060-L-C
15-mL nuclease-free centrifuge tubes	Corning	352096
50-mL nuclease-free centrifuge tubes	Corning	352070
1.5-mL sampling tubes	Eppendorf	3810X, 0030 125.150
10-μL short tips	Watson	120P-204CS
200-μL tips	Watson	1201-705CS
1000-μL tips	FastGene	FG-402ALRS
Low-profile blades	Leica	DB80LS 14035843488
Membrane slides 0.17 PEN (alternative)	Carl Zeiss	415190-9061-000
Membrane slides 1.0 PEN	Carl Zeiss	415190-9041-000
Slide mailer	Bio Medical Science	BC-68951
Silica gel medium granular blue	Wako	192-18305
Terumo syringe 50 mL slip tips (side entrance)	Terumo	SS-50ESZ
Millex-GV syringe filter units, 0.22 μm, PVDF, 33 mm, gamma sterilized	Merck Millipore	SLGVR33RS
Tissue-Tek cryomold	Sakura Finetek Japan	4565
Paper towel (Kimtowel)	Nippon Paper Crecia	61001
DynaMag-2 magnetic stand	Thermo Fisher Scientific	DB12321
384-well Hard-Shell microplate (white well/clear shell)	Bio-Rad	HSP3805
Microseal “B” sealing films	Bio-Rad	MSB1001
Qubit 1× dsDNA HS Assay kit	Thermo Fisher Scientific	Q33230
Qubit assay tubes	Thermo Fisher Scientific	Q32856
Axygen 0.5 mL PCR tubes with flat cap (alternative)	Corning	PCR-05-C
RNase ZAP (optional)	Thermo Fisher Scientific	AM9780
RNase Quiet	Nacalai Tesque	09147-14

## Materials and equipment

### dH_2_O stock

Aliquot distilled water (dH_2_O) into 40 mL, 10 mL and 1 mL aliquots.

***Note:*** To minimize the risk of RNase contamination, we recommend aliquoting dH_2_O in nuclease-free 1.5-mL sampling tubes, 15-mL nuclease-free centrifuge tubes, and/or 50-mL nuclease-free centrifuge tubes and storing them at −20°C. Each aliquot should be used once after thawing.

***Note:*** Avoid using diethylpyrocarbonate (DEPC) and autoclaving for the purpose of preventing RNase contamination, because DEPC may prevent enzymatic reactions for cDNA amplification. Autoclaving may cause contamination of volatile substances.

***Note:*** on storage conditions: The dH_2_O stocks can be stored at −20°C or below for at least one year.

**Table:** 1% Cresyl violet acetate/50% isopropanol

Reagent	Final concentration	Amount
Cresyl violet acetate	1%	100 mg
Isopropanol	50%	5 mL
dH_2_O stock	N/A	5 mL
**Total**	**N/A**	**10 mL**

***Note:*** Mix the solution by inversing on a rotator for at least 16 h at 25°C. Then, remove any insoluble particulates by filtrating through a Millex-GV syringe filter (0.22-μm) attached to a 50 mL Terumo syringe.

***Alternatives:*** Ethanol can be used as a substitute for isopropanol.

***Note:*** on storage conditions: The solution can be stored at 25°C for at least one month.

**Table:** 5% Sodium deoxycholate (SDc) solution

Reagent	Final concentration	Amount
Sodium deoxycholate	5%	100 mg
dH_2_O stock	N/A	2 mL
**Total**	**N/A**	**2 mL**

***Note:*** Prepare this solution in a 50-mL nuclease-free centrifuge tube.

***Note:*** on storage conditions: The solution can be stored at −20°C or below for at least one year. Aliquot 50 μL in 1.5-mL sampling tubes.

**Table:** 100% Triton X-100 stock

Reagent	Final concentration	Amount
Triton X-100	100%	100 μL
**Total**	**N/A**	**100 μL**

***Note:*** Aliquot 100 μL of Triton X-100 in 1.5-mL sampling tubes to minimize the risk of contamination to the original stock. We routinely prepare five tubes of 100% Triton X-100 stock.

***Note:*** on storage conditions: Triton X-100 can be stored at 25°C for at least two years.

**Table:** Betaine (5 M)

Reagent	Final concentration	Amount
Betaine	5 M	2.9275 *g*
dH_2_O stock	N/A	5 mL
**Total**	**N/A**	**5 mL**

***Note:*** on storage conditions: The solution can be stored at −20°C or below for at least one year. Aliquot 100 μL in 1.5-mL sampling tubes.

**Table:** ERCC RNA Spike-In Mix 1 stock solution (1:50)

Reagent	Final concentration	Amount
ERCC RNA Spike-In Mix 1	1:50	1 μL
dH_2_O	N/A	49 μL
**Total**	**N/A**	**50** μ**L**

**CRITICAL:** Use a fresh aliquot of the dH_2_O product specified in the key resources table. Do not use autoclaved or DEPC-treated H_2_O.

**CRITICAL:** Dilute the ERCC RNA Spike-In Mix 1 in a DNA 0.5-mL LoBind tube on ice. Before dilution, chill dH_2_O and all tubes on ice for at least 20 min, then thaw the original solution of ERCC RNA Spike-In Mix1 on ice.

**CRITICAL:** Aliquot 5 μL of the diluted RNA into the DNA 0.5-mL LoBind tubes on ice and immediately store them at −80°C.

***Alternatives:*** As an alternative to the DNA 0.5-mL LoBind tube, the 0.6-mL SnapLock Microtube Maxymum Recovery can be used.

**CRITICAL:** After thawing, ensure the single use of the RNA aliquots.

***Note:*** on storage conditions: The solution can be stored at −80°C or below for at least one year.

**Table:** ERCC RNA Spike-In Mix 1 stock solution (1:5000)

Reagent	Final concentration	Amount
ERCC RNA Spike-In Mix 1 stock solution (1:50)	1:5000	1 μL
dH_2_O	N/A	99 μL
**Total**	**N/A**	**100** μ**L**

**CRITICAL:** Use a fresh aliquot of dH_2_O. Do not use autoclaved or DEPC-treated H_2_O.

**CRITICAL:** Aliquot 5 μL of the diluted RNA in the DNA 0.5-mL LoBind tubes or 0.6-mL SnapLock Microtube Maxymum Recovery and immediately store them at −80°C.

**CRITICAL:** After thawing, ensure the single use of the RNA aliquots.

***Note:*** on storage conditions: The solution can be stored at −80°C or below for at least one year.

Troubleshooting 1.

**Table:** Qiagen protease (900 mAU/mL) (Optional)

Reagent	Final concentration	Amount
Qiagen protease (7.5 AU, lyophilized)	900 mAU/mL	7.5 AU
dH_2_O stock (10 mL)	N/A	8.333 mL
**Total**	**N/A**	**8.333 mL**

***Note:*** Dissolve all the lyophilized protease by adding dH_2_O directly to the original tube.

***Note:*** on storage conditions: The solution can be stored at −20°C or below for at least one year. Aliquot 50 μL of Qiagen protease solution into Protein 1.5-mL LoBind tubes.

## Step-by-step method details

### Part 1. Sectioning of frozen ovaries

**Timing: 2 h**

**Figure 4:**
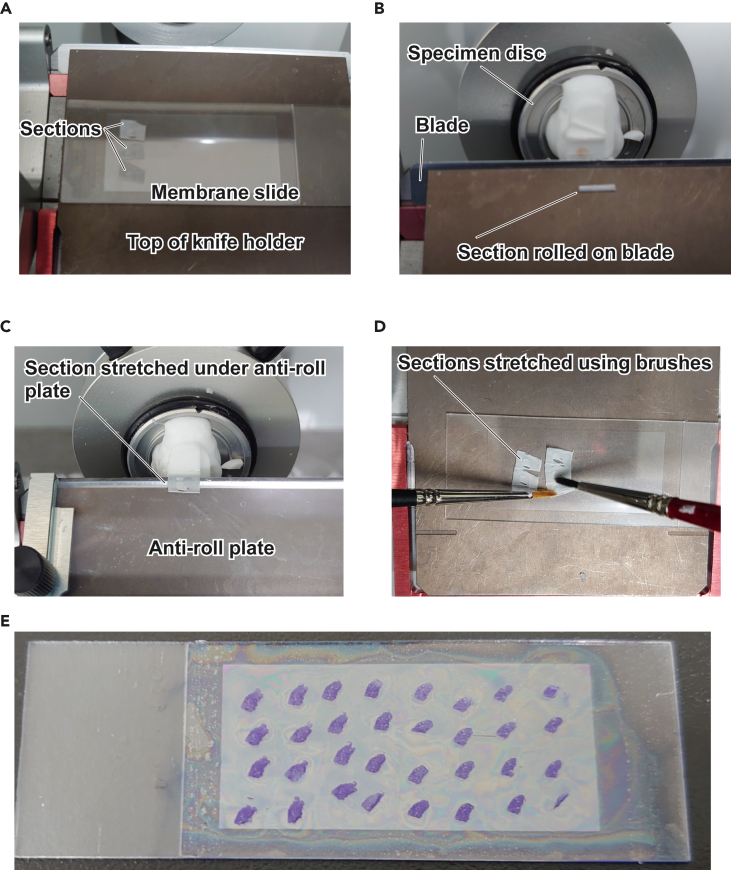
Sectioning of frozen ovaries (A) Frozen sections successfully mounted on a membrane slide. (B) Frozen section rolled on the blade. (C) Section stretched under the anti-roll plate. (D) Section stretched using brushes. (E) Membrane slide mounted with ovarian sections stained with Cresyl violet.

In this step, the frozen mouse ovary is sectioned. The resulting sections are mounted on membrane slides (Figure 4).

1. Place a Membrane Slide 1.0 PEN in the cryochamber of a CM1860UV cryomicrotome at −20°C.

***Note:*** The cellular contents within a section are directly exposed to the environment and are highly susceptible to RNase contamination. To minimize this risk, we routinely clean the cryochamber with RNase Quiet or RNase ZAP and 70% ethanol before use, and additionally sterilize it using UV for 15 min–1 h.

2. Section the frozen ovaries using a low-profile blade at a thickness of 15 μm.

**CRITICAL:** Use a new blade to prevent RNase contamination.

***Note:*** The optimal thickness of sections may depend on the tissue and the specific cell types of interest. In our experience, the optimal thickness for frozen sections of mouse ovary, skeletal muscle, and brain typically falls within the range of 8–15 μm. For formalin-fixed paraffin-embedded (FFPE) murine ovarian sections, the recommended thickness is typically 8 μm.

3. Gently stretch the thin sections on the membrane slide and adhere them by transferring to 25°C.

***Note:*** To prevent repeated freeze-thaw cycles of the sections before drying, move the slide to 25°C after placing all the sections on the membrane slides.

***Alternatives:*** The slide glass can be warmed locally with a finger to adhere sections one by one.

***Alternatives:*** According to the manufacturer’s instructions (Zeiss, LCM Protocols – RNA handling), the adhesiveness of the membrane slides can be enhanced by irradiating with UV light at 254 nm for 30 min and by coating with 0.1% Poly-L-Lysine. Excess Poly-L-Lysine may be removed by washing the slide with dH_2_O. In our experience, FFPE sections may need these treatments for robust adhesion.

Troubleshooting 2.

4. Dry the sections at 25°C for 0.5 h–1 h.

**CRITICAL:** Cover the slides with aluminum foil to avoid RNase contamination.

### Part 2. Staining of frozen sections

**Timing: 5 min**

**Figure 5:**
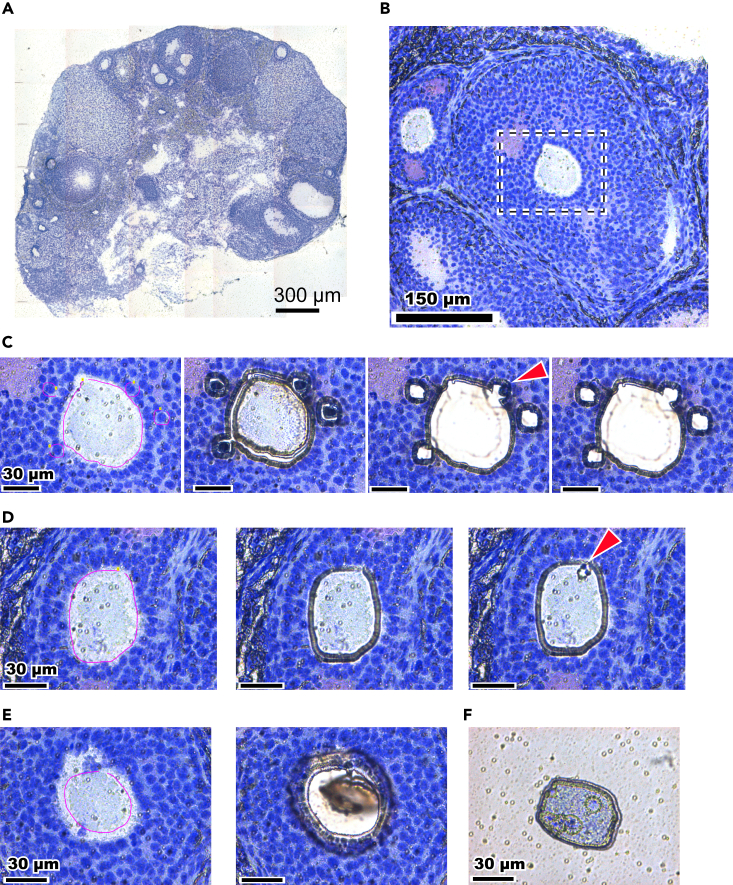
Examples of LCM of oocytes and granulosa cells from ovarian sections (A) A mouse ovarian section mounted on a membrane slide stained with Cresyl violet. Microscopic images of sections were tiled to reconstruct the whole ovary. Bar: 300 μm. (B) Close-up view of a follicle. The area in the dashed square shows oocytes and granulosa cells targeted by the LCM and is further magnified in panel C. Bar: 150 μm. (C) Successful single-cell LCM of an ovarian section. Shown are oocyte and granulosa cells superimposed with guidelines for laser cutting (left), the results of joint cutting (2nd left), and results of the first (2nd right) and second LPCs (right). The red arrowhead indicates a joint-cut granulosa cell that failed in the first LPC but was successfully captured by the second LPC. Bar: 30 μm. (D) Failed laser cutting of an oocyte. Shown are an oocyte superimposed with a guide line (left), an incomplete joint-cut (2nd left), and a result of failed LPC (right). The red arrowhead indicates a mark of LPC. Bar: 30 μm. (E) Failed LPC of an oocyte. Shown are an oocyte superimposed with a guide line for laser cutting (left) and a result of failed LPC after joint cutting (right). Bar: 30 μm. (F) An oocyte that fell on the membrane slide after LPC and failed to be captured in the tube cap. Bar: 30 μm.

In this step, the frozen ovarian sections are stained with Cresyl violet acetate (Figure 5).

5. Place the membrane slides mounted with the frozen sections on a paper towel (Kimtowel) at 25°C.

***Note:*** The paper towel is expected to absorb excess staining solution in the subsequent steps.

6. Apply 500 μL of 0.1%–1% Cresyl violet acetate/50% isopropanol to the frozen sections on the membrane slide and allow it to settle for 30 s at 25°C.

***Note:*** The concentration of Cresyl violet should be adjusted according to the tissue types and fixation methods. For snap-frozen murine ovarian sections, we routinely use 1% Cresyl violet/50% isopropanol for LCM.

***Note:*** In this step, tissue sections are fixed with 50% isopropanol contained in the staining solution.

7. Discard the excess staining solution by tilting the membrane slide over the paper towel.

***Optional:*** Immediately after Step 7, the sections can be additionally stained with Eosin Y Stain Solution (0.17% Eosin Y, 60% ethanol) for 50 sec at 25°C. Discard the excess staining solution by tilting the membrane slide over the paper towel. Eosin Y stains cytosol and extracellular collagen fibers. This additional staining does not significantly affect cDNA amplification.

8. Wash the slide once with 50%–100% isopropanol.

***Note:*** Determine the isopropanol concentration and number of washings per tissue type, fixative, and staining method. If necessary, try additional washing, but avoid excess washing. In addition, be aware that a high isopropanol concentration may result in over-drying, leading to the formation of cracks in the sections. We recommend trying 50% isopropanol for this step.

***Alternatives:*** 50%–100% ethanol can be used as a substitute for isopropanol.

9. Allow the sections to air dry for 1 h at 25°C.

**Figure 6:**
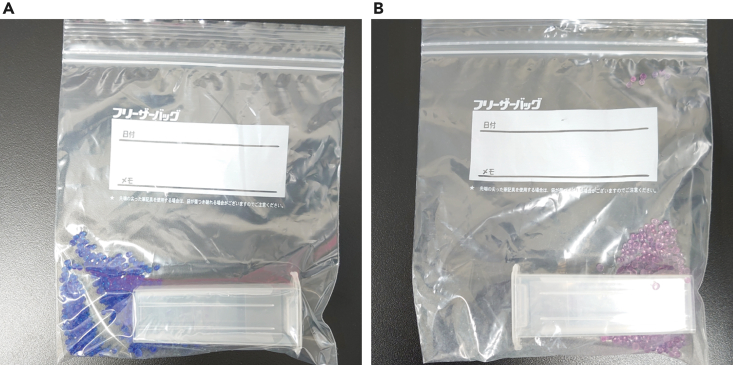
Freeze-stock of membrane slides (A) Ziploc bag containing a slide mailer with fresh, blue silica gel. (B) Ziploc bag containing a slide mailer with expired, pink silica gel.

***Note:*** Determine the appropriate drying time up to 24 h per tissue type and fixative. Avoid over-drying.

**Pause point:** The sections can be stored at −80°C for at least 6 months. For storage, place the membrane slides mounted with the sections in a slide mailer, and then put them into a Ziploc bag. To prevent dew condensation on the slides, place 5 *g* of Silica Gel Medium Granular Blue into the Ziploc bag containing the slides. If the stored sections will be used for LCM, return the Ziploc bag to 25°C in a stepwise manner: −20°C for at least 10 min, 4°C for 10 min, then 25°C (Figure 6). The silica gel may be added to the Ziploc bag on taking it out from −80°C storage. If the color of the silica gel changes from blue to pink, replace it with new silica gel. Preventing dew condensation is necessary because internal RNases may be activated in liquid water.

Troubleshooting 3.

### Part 3. Laser capture microdissection

**Timing: 3 h**

In this step, cells of interest and/or regions of interest (cells/ROIs) are identified through microscopic inspection of the stained ovarian sections and are isolated using a PALM MicroBeam 4 (MB4) laser microdissection system (Carl Zeiss). These cells/ROIs are captured directly in cell lysis buffer contained in the caps of single flat-top 200-μL PCR tubes.

***Alternatives:*** This protocol focuses on DRaqL-Smart-seq2 for alcohol-fixed frozen sections. However, when the sections are fixed with formalin, DRaqL should be combined with thermolabile protease treatment (DRaqL-Protease-Smart-seq2) (see Step 12, Steps 21–24 and Figure 1).

***Alternatives:*** We typically use a Carl Zeiss PALM MB4 system for LCM. However, DRaqL might be adapted to other microdissection systems/methods for tissue sections, such as laser microdissection systems from Leica and Arcturus, a multiplexed laser-based device,^9^ vacuum pulse-assisted systems (Unipick, NDX-Instruments), and patch pipette-based method.^10^

10. Determine LCM parameters (i.e., cutting energy, laser pulse catapulting (LPC) energy, joint width, and speed) using surplus areas in the sections.

***Note:*** The LCM parameters should be determined using objective lens that will be actually used for isolating cell/ROIs: an objective lens 20×, 40×, or 63×.

***Note:*** For isolating oocytes and granulosa cells in alcohol-fixed mouse ovarian sections, we identified the optimal parameters for the objective lens 63× as follows: cutting energy at 40%–60%, LPC energy at 30%, joint width at 5 μm, and speed at 40%.

***Note:*** Stronger dye staining can enhance laser cutting efficiency.

***Optional:*** An objective lens 100× can also be used for LCM. In this case, a thin Membrane Slide 0.17 PEN (0.17-mm thickness) would be required due to the short focal distance of this lens.

11. Cut the cells/ROIs using the Joint-Cut mode.
  a. Identify oocytes and granulosa cells of interest in the ovarian sections.
  b. Draw guide lines for laser cutting by encircling the targeted cells/ROIs (Figure 5).
  c. Cut all the targeted cells/ROIs using the Joint-Cut mode with the parameters determined in Step 10 (i.e., the parameters for cutting energy, joint width, and speed) (Figure 5).

***Note:*** After this step, the cells/ROIs remain within the sections due to the joints created during the joint cutting.

***Note:*** Use an appropriate objective lens for LCM depending on the target tissues and cells/ROIs. We routinely use an objective lens 40× or 63× for murine ovarian sections.

***Note:*** Given the 8–15 μm thickness used for the murine ovarian sections, which is similar to the diameter of a single granulosa cell, it is unlikely that two or more entire cells will be collected simultaneously into a single tube. In addition, this range of thickness will not cause defects for the RNA-seq results of oocytes, which have diameters larger than the thickness of the sections, as demonstrated in our previous study.^1^

Troubleshooting 4 and Troubleshooting 5.

12. Prepare sufficient amount of cell lysis buffer and ERCC RNA Spike-In Mix 1 (1:500,000) for all the joint-cut cells on ice in 1.5-mL sampling tubes.

**CRITICAL:** Before preparing the cell lysis buffer, ensure successful joint cutting of all cells/ROIs.

***Note:*** The protocol below is optimized for single oocytes and granulosa cells. However, ROIs composed of up to approximately 300 granulosa cells can also be applied to this protocol with only minor modifications (see Alternative note at Step 30).

Troubleshooting 6.

**Table:** ERCC RNA Spike-In Mix 1 (1:500,000)

Reagent	Final concentration	Amount
ERCC RNA Spike-In Mix 1 stock solution (1:5000)	1:500,000	1 μL
dH_2_O stock (1 mL)	N/A	99 μL
**Total**	**N/A**	**100 μL**

**CRITICAL:** Before use, chill dH_2_O on ice for at least 20 min.

***Note:*** Prepare this solution on ice immediately before use.

***Note:*** Ensure single use of the ERCC RNA Spike-In Mix 1 stock solution (1:5000) after thawing.

**Table:** Cell lysis buffer

Reagent	Final concentration	Amount
5× SuperScript II buffer	0.46875×	0.6 μL
Oligo dT VN IL (100 μM)	1.5625 μM	0.1 μL
dNTP mix (25 mM each)	3.125 mM	0.8 μL
Recombinant RNase inhibitor (40 U/μL)	1.5625 U	0.25 μL
DTT (100 mM)	7.8125 mM	0.5 μL
MgCl_2_ (1 M)	9.375 mM	0.06 μL
Betaine (5 M)	1.5625 M	2 μL
ERCC RNA Spike-In Mix 1 (1:500,000)	1:200,000,000	0.16 μL
SDc (5%)	0.3125%	0.4 μL
dH_2_O	N/A	1.53 μL
**Total**	**N/A**	**6.4 μL**

**CRITICAL:** Chill all reagents except for ERCC RNA Spike-In Mix 1 and Recombinant RNase inhibitor on ice for at least 20 min. Immediately before use, add the RNase inhibitor, then RNAs to the mixture.

***Note:*** Increasing the amount of RNase inhibitor may improve the suppression of contaminated RNase activity. For example, in our experience, incorporating up to 1.2 μL of RNaseOUT Recombinant Ribonuclease Inhibitor resulted in enhanced cDNA amplification using DRaqL-SC3-seq.^1^

***Note:*** In this step, different IL index sequences in the reverse transcription primers (Oligo dT VN IL) are employed to label different cells. The IL indexes allow pooling of cDNA after the first-strand synthesis (Step 29) (Figure 1) or before the library preparation of the 3′-end sequencing (Steps 51) (Figure 2). However, the same IL index can be used to label cDNA samples from different cells at Step 12, as long as their sequencing libraries will be labeled with different P5 and P7 indexes at Step 61 (see Figure 3). Consequently, at this step, we routinely prepare 12 tubes each containing 100 μL of cell lysis buffer with different IL indexes. This amount of cell lysis buffer is sufficient for cDNA amplification from 10–12 cells/ROIs, each labeled with an IL index, allowing 120–144 cells/ROIs in total to be captured for RNA-seq.

***Note:*** The IL indexes label 3′-ends. Therefore, short-read, full-length mRNA sequencing does not allow the pooling of cDNA (see also the Alternative note at Step 61).

***Note:*** The sequences of the IL indexes in this study are the same as those used in the SMART-seq v4 3′DE Kit (Takara) (Table S1).

***Note:*** If the total RNA amount contained in target cells is expected to be considerably less than 10 pg, we recommend reducing the amount of primers for reverse transcription (Oligo dT VN IL) (Step 12) and template switching (N-template-switching oligo) (Step 24) to prevent the production of byproducts, as previously noted.^11^ In our experience, Oligo dT VN IL and N-template-switching oligo can be reduced to 1/4 of the original amount without a significant decrease in cDNA amplification efficiency.

***Alternatives:*** Unique molecular identifiers (UMIs) can also be incorporated into Oligo dT VN IL (N-Oligo dT VN IL UMI; see Table S1) without a significant decrease of cDNA amplification efficiency.

***Alternatives:*** In our experience, other denaturing detergents, such as sodium dodecyl sulfate (SDS), sodium N-lauroylsarcosinate, and sodium cholate, can be used as substitutes for SDc at concentrations of 0.15%–0.25% (data not shown). In addition, other quenching detergents, such as polyoxyethylene (23) lauryl ether (Brij L23, Brij 35) and Tween 20, can also be used as substitutes for Triton X-100 at the same concentration (final 7.6%). However, there may be a trade-off between lysis and cDNA amplification efficiencies because stronger denaturing agents (e.g., 0.25% SDS) may reduce cDNA amplification efficiency.^1^

***Alternatives:*** If the sections have been fixed with formalin, follow the procedures of DRaqL-Protease-Smart-seq2 in Steps 12 and 21–24 (Figure 1). For DRaqL-Protease-Smart-seq2, prepare a modified cell lysis buffer in this step (Step 12), by incorporating treatment of a thermolabile protease (Qiagen protease) while excluding the ERCC RNA Spike-In Mix 1 (cell lysis buffer for DRaqL-Protease-Smart-seq2). The ERCC RNA Spike-In Mix 1 will be added after heat inactivation of protease (see Alternative note at Steps 21–24).

**Table:** Cell lysis buffer for DRaqL-Protease-Smart-seq2 (alternative)

Reagent	Final concentration	Amount
5× SuperScript II buffer	0.46875×	0.6 μL
Oligo dT VN IL (100 μM)	1.5625 μM	0.1 μL
dNTP mix (25 mM each)	3.125 mM	0.8 μL
Recombinant RNase inhibitor (40 U/μL)	1.5625 U	0.25 μL
DTT (100 mM)	7.8125 mM	0.5 μL
MgCl_2_ (1 M)	9.375 mM	0.06 μL
Betaine (5 M)	1.5625 M	2 μL
Qiagen protease (900 mAU/mL)	45 mAU/mL	0.32 μL
SDc (5%)	0.3125%	0.4 μL
dH_2_O	N/A	1.37 μL
Total	N/A	6.4 μL

13. Apply 6.4 μL of cell lysis buffer into the cap of a single flat-top 200-μL PCR tube (Figure 7).

**Figure 7:**
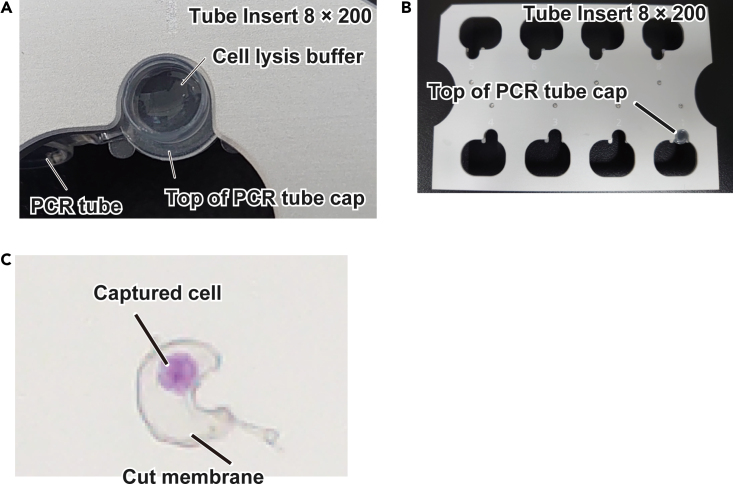
Capturing cells/ROIs in cell lysis buffer (A) A tube cap containing cell lysis buffer, set in the tube insert. (B) A long shot of the tube insert shown in A. (C) A Cresyl violet-stained culture cell on a PEN membrane captured in cell lysis buffer after joint cutting and LPC.
14. Place the tube cap containing cell lysis buffer (product of Step 13) on the Tube Insert 8 × 200 equipment of the PALM MB4 laser microdissection system.
15. Perform LPC using the parameters determined in Step 10 to capture the joint-cut cells/ROIs (products of Step 11) in the cell lysis buffer in the cap.
16. Collect the captured cells/ROIs in the PCR tube.
  a. Close the cap promptly after the LPC.
  b. Tap the tube ten times and then immediately centrifuge it at 5000 × *g* for 10 s.
  c. Place the tube on ice.

Troubleshooting 7.

17. Freeze the tube in liquid nitrogen and store it at −80°C or proceed directly to Step 18.

**Pause point:** The frozen samples can be stored at −80°C for at least one week.

18. Repeat Steps 13–17 to capture all the cells/ROIs.

**CRITICAL:** During Steps 13–16, ensure that the cell lysis buffer completely covers the bottom of the flat cap. Evaporation of the buffer will dry the cap bottom and prevent efficient capture of cells/ROIs.

***Alternatives:*** The optimal volume of cell lysis buffer may depend on the amount of evaporation during Steps 13–16. In our experience, up to 10 μL of cell lysis buffer can be used without a significant decrease in cDNA amplification efficiency. If the volume of cell lysis buffer is scaled up, increase the volume of downstream reaction mixtures proportionally (quenching buffer [Steps 19 and 24], template-switching mixture [Steps 20 and 25], and SeqAmp PCR mixture [Steps 27 and 29]).

***Note:*** After testing various types of PCR tubes with flat-top caps, we have identified the single flat-top 200-μL PCR tubes shown in the key resources table as the optimal choice. This selection is based on the narrow top of the cap, which leads to a relatively low evaporation rate of cell lysis buffer.

***Optional:*** Sections left after LCM can be stored at −80°C for at least 6 months. For storage and reuse of sections, see the Pause point at Step 9.

### Part 4. cDNA amplification

**Timing: 3.5 h**

In this step, cells/ROIs isolated from alcohol-fixed sections are lysed by SDc, which is then quenched by an excess amount of Triton X-100. These procedures seamlessly combine efficient cell lysis and cDNA amplification.

***Note:*** The denaturing activity of SDc is weakly retained even after quenching. Thus, we selected enzymes active during these conditions. In particular, reverse transcriptase activity of SuperScript II is inhibited, while its template-switching activity is retained. In contrast, SuperScript III, having no template-switching activity, retains sufficient reverse transcriptase activity. This justifies the use of both SuperScript II and III in this step. Alternatively, as noted in Step 20, another reverse transcriptase, PrimeScript II, shows both sufficient reverse transcriptase and template-switching activities under these conditions and can be used as a substitute for both Superscript II and III.

***Alternatives:*** For formalin-fixed sections, follow the procedure of DRaqL-Protease-Smart-seq2 (see Alternative notes for Steps 21–24 and Figure 1).

19. Prepare quenching buffer at 25°C.

**Table:** Quenching buffer

Reagent	Final concentration	Amount
5× SuperScript II buffer	2.5×	1.4 μL
100% Triton X-100 stock	25%	0.7 μL
BSA (20 mg/mL)	5 mg/mL	0.7 μL
**Total**	**N/A**	**2.8 μL**

***Note:*** As 100% Triton X-100 is highly viscous, pipette it carefully to measure the correct volume. Ensure thorough mixing by repetitive tapping.

***Note:*** Avoid chilling the quenching buffer.

***Optional:*** To reduce the viscosity, incubate the 100% Triton X-100 stock, 5× SuperScript II buffer, and BSA at 50°C immediately before use. Then, mix these pre-warmed reagents at 25°C.

20. Prepare the template-switching mixture on ice.

**Table:** Template-switching mixture

Reagent	Final concentration	Amount
N-template-switching oligo (100 μM)	6.25 μM	0.1 μL
SuperScript II (200 U/μL)^∗^	31.25 U	0.25 μL
SuperScript III (200 U/μL)^∗^	31.25 U	0.25 μL
Recombinant RNase inhibitor (40 U/μL)^∗^	5 U	0.2 μL
dH_2_O	N/A	0.8 μL
**Total**	**N/A**	**1.6 μL**

^∗^Add these protein components just before use.

**CRITICAL:** Prepare the template-switching mixture (Step 20) and SeqAmp PCR mixture (Step 27) without protein components (SuperScript II, SuperScript III, recombinant RNase inhibitor, or SeqAmp DNA polymerase). Chill these protein-free mixtures on ice for at least 20 min. Then, add the proteins immediately before use at Step 25 (template-switching mixture) and Step 29 (SeqAmp PCR mixture).

***Note:*** To simplify the process, just before applying SuperScript II and SuperScript III to the template-switching mixture, mix these enzymes at a 1:1 ratio in a protein 0.5-mL LoBind tube on ice (i.e., apply 0.5 μL of the 1:1 mixture to the template-switching mixture).

***Alternatives:*** 0.5 μL of PrimeScript II can be used as a substitute for both SuperScript II and SuperScript III without decreasing the cDNA amplification efficiency.

***Alternatives:*** As mentioned above (see Note on cell lysis buffer at Step 12), if the amount of RNA is expected to be small, the amount of N-template-switching oligo may be reduced.

21. Briefly centrifuge the PCR tubes containing cells/ROIs (products of Step 18) to collect the contents at the bottom, then place them on ice.
22. Place the tubes into a thermal cycler (Eppendorf Mastercycler nexus or equivalent) with a heated lid and incubate them at 72°C for 6 min for cell lysis.
23. Put the tubes (product of Step 22) on ice for 1 min and centrifuge briefly to bring the contents to the bottom of the tube. Then, put them back on ice.
24. Add quenching buffer to each tube.
  a. Apply 2.8 μL of quenching buffer onto the inner wall of the tube.
  b. Centrifuge the tube briefly and place it back on ice.
  c. Thoroughly mix the liquid by tapping, and promptly put the tube back on ice.
    ***Note:*** After the addition of quenching buffer to the cell lysis buffer, the concentrations of Triton X-100 and BSA in the mixture are 7.6% and 0.15%, respectively.
    ***Note:*** Because the quenching buffer is highly viscous, ensure thorough mixing of the liquid by visual inspection.
    Troubleshooting 8.
    ***Alternatives:*** For formalin-fixed sections, follow the procedure of DRaqL-Protease-Smart-seq2 as an alternative to Steps 21–24 (Figure 1). First, confirm that the cell lysis buffer for DRaqL-Protease-Smart-seq2 has been used in Step 12. Then, replace Steps 21–24 with the following procedures (i–viii):
    i. Prepare the ERCC mixture (1:2,500,000) on ice (0.16 μL ERCC RNA Spike-In Mix 1 [1:500,000], 0.64 μL dH_2_O; total 0.8 μL).
    ii. Briefly centrifuge the tubes containing the samples (products of Step 18) and place them on ice.
    iii. Incubate the tubes at 50°C–60°C for 10 min–24 h for protease treatment using a pre-heated thermal cycler, followed by inactivation of the protease at 80°C for 15 min.
      ***Note:*** The temperature and duration of protease treatment should be determined for different samples and fixation conditions. For example, efficient cell lysis of sections from FFPE mouse ovaries may require protease treatment at 60°C for 10 min. Note the potential trade-off due to RNA degradation or hydrolysis by the prolonged protease treatment.
    iv. Place the tubes on ice for 1 min and briefly centrifuge.
    v. Add 0.8 μL of the ERCC mixture (1:2,500,000) (product of Step i) to each tube on ice.
    vi. Add 2.8 μL of quenching buffer (product of Step 19) to each tube on ice and mix it thoroughly (see Step 24).
    vii. Incubate the tubes at 72°C for 90 s using a pre-heated thermal cycler.
    viii. Place the tubes on ice for 1 min and centrifuge briefly. Then, proceed to Step 25.
25. Add 1.6 μL of the template-switching mixture to each tube and mix it gently by tapping. Spin it down briefly and then place the tubes on ice.

**CRITICAL:** Ensure that the template-switching mixture, excluding the protein components (SuperScript II, SuperScript III, and recombinant RNase inhibitor), has been prepared on ice at Step 20. Add these protein components to the protein-free mixture immediately before use in Step 25.

26. Place the tubes into a thermal cycler with a heated lid and run the cycling RT program for DRaqL-Smart-seq2 to conduct the first-strand cDNA synthesis.

**Table:** Cycling RT program for DRaqL-Smart-seq2

Steps	Temperature	Time	Cycles
Initial annealing	42°C	90 min	1
Reverse transcription	50°C	2 min	10 cycles
Annealing	42°C	2 min	
Inactivation	70°C	15 min	1
Hold	4°C	infinite

**CRITICAL:** Set the temperature ramp at 1°C/sec during the entire protocol.

27. During the cycling RT program, prepare the SeqAmp PCR mixture on ice.

**Table:** SeqAmp PCR mixture

Reagent	Final concentration	Amount
2× SeqAmp buffer	1.67×	12.5 μL
N-IS PCR primer (100 μM)	0.33 μM	0.05 μL
SeqAmp DNA polymerase	N/A	0.5 μL
dH_2_O stock (1 mL)	N/A	1.95 μL
**Total**	**N/A**	**15 μL**

**CRITICAL:** Mix all reagents except for SeqAmp DNA polymerase, then chill them on ice for 20 min. At Step 29, add SeqAmp DNA polymerase to the protein-free mixture immediately before use.

28. When Step 26 is complete, place the tubes on ice.
29. Add 15 μL of SeqAmp PCR mixture to each tube and mix gently by tapping. Then, spin down the tube briefly and place it on ice.

**CRITICAL:** Add the SeqAmp DNA polymerase to the protein-free SeqAmp PCR mixture (product of Step 27) immediately before use.

***Alternatives:*** If only 3′-end sequencing is planned, it is permissive to pool up to 6 samples of the first-strand cDNAs labeled with different IL indexes (product of Step 26) (Figure 1). In this case, increase the volume of the SeqAmp PCR mixture proportionally. This pooling can simplify the procedures in Steps 29 and 30.

30. Place the tubes into the thermal cycler with a heated lid and run the thermal cycling program for SeqAmp.

**Table:** Thermal cycling program for SeqAmp

Steps	Temperature	Time	Cycles
Initial denaturation	95°C	1 min	1
Denaturation	98°C	10 s	20 cycles
Annealing	65°C	30 s	
Extension	68°C	6 min	
Final extension	72°C	10 min	1
Hold	10°C	infinite

***Note:*** At this step, the total sample volume is 25.8 μL.

***Alternatives:*** If the amount of RNA is expected to be significantly larger than 10 pg, the number of PCR cycles may be reduced. For example, we typically employ 18 cycles for single oocytes and bulk ROIs of granulosa cells (∼300 cells).

**Pause point:** The samples can be stored at 4°C or below for at least 1 day.

Troubleshooting 9.

### Part 5. Purification of cDNA

**Timing: 1 h**

In this step, the amplified cDNA is purified using AxyPrep MAG PCR Clean-up Kit.

31. Prepare 70% ethanol in a 50 mL nuclease-free centrifuge tube and set up a DynaMag-2 magnetic stand.

***Alternatives:*** Magnetic stands from any other manufacturer may be used in this step.

**Table:** 70% Ethanol

Reagent	Final concentration	Amount
Ethanol	70%	14 mL
dH_2_O	N/A	6 mL
**Total**	**N/A**	**20 mL**

32. Apply 20 μL paramagnetic bead solution of AxyPrep MAG PCR Clean-up Kit (∼0.8× volume of the cDNA sample at Step 30) into 1.5-mL sampling tubes at 25°C.

***Note:*** The amount of reagent is optimized for purifying cDNA (larger than 300 bps) and removing primer–dimer byproducts smaller than 300 bps.

***Alternatives:*** Any other solid phase reversible immobilization (SPRI) paramagnetic beads product, such as AMPure XP reagent, can be used in this step. However, the volume of the beads suspension may need optimization. In our experience, AMPure XP reagent can be used in the same manner as the AxyPrep MAG PCR Clean-Up Kit.

33. Add each cDNA sample (product of Step 30) to a 1.5-mL sampling tube containing the AxyPrep MAG PCR Clean-up beads (product of Step 32), mix them by pipetting or vortexing, and incubate the suspensions at 25°C for 5 min.
34. Place the tubes on the magnetic stand for 3 min and allow the solution to clear.
35. Carefully remove the supernatant using a micropipette with a 200-μL tip.
36. Add 200 μL of 70% ethanol to each tube, mix it by pipetting or vortexing, and incubate it for 30 s at 25°C.
37. Place the tubes onto the magnetic stand for 3 min until the solution clears, then remove the supernatant.
38. Repeat Steps 35–37 once.
39. Remove residual ethanol in the tubes.
  a. Briefly centrifuge the tubes to collect remaining ethanol at the bottom.
  b. Place the tubes on the magnetic stand.
  c. Remove all residual ethanol using a micropipette.

**CRITICAL:** Thoroughly remove the ethanol from the tube bottom to prevent its carryover to subsequent processes.

40. To elute DNA, add 50 μL of dH_2_O to each tube and resuspend the magnetic beads by vortexing.
41. Place the tubes on the magnetic stand for 3 min to allow the solution to clear. Transfer the supernatant containing cDNA into new tubes.

**Pause point:** The purified cDNAs can be stored at −20°C or below for at least one year.

### Part 6. Quality control of cDNA: Real-time PCR

**Timing: 3 h**

This step is optional, but we recommend evaluating the quality of cDNA samples (product of Step 41) by performing real-time PCR for housekeeping genes (e.g., *Arbp*) and/or spike-in ERCC RNAs (e.g., *ERCC096*, *ERCC042*).

42. Prepare the following primers at a concentration of 8 μM.

**Table:** Examples of primers for real-time PCR

Gene name	Forward primer	Reverse primer
*ERCC00096* (spike-in)	5′-GATCCCGGAAGATACGCTCTAAG-3′	5′-CGCAGGTTGATGCTTCCAATAAA-3′
*ERCC00042* (spike-in)	5′-GTGGTCTGCATAAGGGTAGAGAG-3′	5′-GCTTTGTCTTTAAACGCTCACCT-3′
*Arbp* (mouse)	5′-CAAAGCTGAAGCAAAGGAAGAG-3′	5′-AATTAAGCAGGCTGACTTGGTTG-3′

***Note:*** The quality of these primers is OPC or desalting grade.

***Note:*** These primers can be stored at −20°C or below for at least one year.

43. Dilute cDNAs at a 1:40 ratio with dH_2_O.
44. Prepare real-time PCR master mix for each primer set.

***Note:*** In this step, we routinely use 2×Power SYBR Green PCR Master Mix and a CFX Opus 384 Real-Time PCR system. However, any other kits and real-time PCR systems can be used.

**Table:** Real-time PCR master mix

Reagent	Final concentration	Amount
2×Power SYBR Green PCR Master Mix	1.25×	5 μL
Forward primer (8 μM)	0.25 μM	0.25 μL
Reverse primer (8 μM)	0.25 μM	0.25 μL
dH_2_O stock (1 mL)	N/A	2.5 μL
**Total**	**N/A**	**8 μL**

45. Apply 2 μL of the diluted cDNAs to one well of a 384-well Hard-Shell Microplate (white well/clear shell), and then add 8 μL of the real-time PCR master mix on ice.

***Note:*** It is recommended that at least two replicates are prepared for each primer set per sample.

46. Perform real time PCR.
  a. Seal the 384-well microplate with a Microseal ‘B’ sealing film.
  b. Centrifuge the microplate at >1000 × *g* for 1 min to collect the contents at the bottom.
  c. Place the microplate at a CFX Opus 384 Real-Time PCR system.
  d. Run the thermal cycling program to perform real-time PCR.

**Table:** Thermal cycling program to perform real-time PCR

Steps	Temperature	Time	Cycles
Initial denaturation	95°C	10 min	1
Denaturation	95°C	15 s	40 cycles
Annealing and extension	60°C	60 s	
Melt curve	65°C–90°C (0.5°C/cycle)	10 s	50 cycles

**Figure 8:**
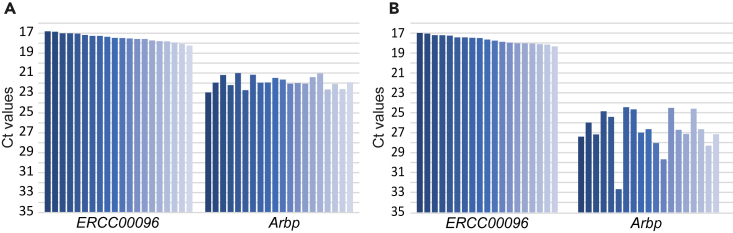
Real-time PCR for specific genes in single-cell cDNAs from sections (A) Bar graphs showing *Ct* values for real-time PCR using successfully amplified cDNAs from single granulosa cells isolated from a frozen ovarian section. (B) Bar graphs showing *Ct* values for real-time PCR using unsuccessfully amplified cDNAs from single granulosa cells isolated from a frozen ovarian section. The unsuccessful cell lysis in (B) is suggested by the variable *Ct* values of *Arbp*, while the consistent *Ct* values of spike-in RNA indicate the success of the cDNA amplification itself.

47. Compare cycle threshold (C_t_) values among samples (see expected outcomes and Figure 8).

### Part 7. Quality control of cDNA: Quantification of cDNA amount

**Timing: 1 h**

This step is optional. The total amount of cDNAs may be quantified.

***Note:*** For this step, we use a Qubit 4 Fluorometer and Qubit 1× dsDNA HS Assay kit. However, any other kits and equipment for DNA quantification can be used for this step.

***Note:*** In our experience, typical yields of cDNA samples from single oocytes and granulosa cells in sections with 8–15 μm thickness after a 20-cycle PCR falls within the ranges of 25–50 ng and 5–10 ng, respectively. From 10-pg total RNA purified from murine embryonic stem cells (mESCs), we typically obtain 10–25 ng total cDNA (See also expected outcomes).

48. Prepare a sufficient number of Qubit assay tubes for the cDNA samples (product of Step 41) and two standards (Standard #1 and #2 contained in the Qubit 1× dsDNA HS Assay kit).

***Alternatives:*** Other 0.5-mL clear polypropylene tubes with flat caps can be used for this step (e.g., Axygen 0.5 mL PCR tubes with flat cap).

49. Prepare mixtures of cDNA samples and Qubit assay reagents.
  a. Dispense 199 and 190 μL of the Qubit 1× dsDNA HS Assay Reagent into the Qubit assay tubes for the cDNA samples and standards, respectively.
  b. Apply 1 μL of cDNA samples and 10 μL of standards to the Qubit assay reagent within the tubes.
  c. Thoroughly vortex the mixture.
  d. Incubate the tubes at 25°C for 2 min.
50. Put the tubes into the Qubit 4 Fluorometer and quantify the DNA amount according to the manufacturer’s instructions.^12^

### Part 8. Library preparation for the 3′-end sequencing

**Timing: 1.5 h**

In this step, DNA libraries for the 3′-end sequencing are prepared from amplified cDNAs (products of Step 41). This process involves tagmentation (Steps 51–59) followed by PCR with P5 and P7 index sequences (Tn Forward PCR primer and Reverse PCR primer) (Steps 60–63).

***Note:*** cDNAs labeled with different IL index sequences are pooled together in Step 51.

***Note:*** In this protocol, we use the Nextera XT DNA Library Prep Kit following the manufacturer’s instructions^13^ and the protocol of the library construction module in the SMART-Seq v4 3′ DE Kit.^14^

***Alternatives:*** Libraries for short-read, full-length sequencing can be generated using another set of primers in Steps 60–63 (Tn Forward PCR primer and Tn Reverse PCR primer; see Table S1), as explained in Alternative note at Step 61 (Figure 2). In this case, the initial pooling of cDNAs at Step 51 should be omitted because the IL indexes label only the 3′-end of cDNA (see Alternative notes in Steps 12 and 51).

51. Mix cDNA samples labeled with different IL indexes.

**CRITICAL:** Ensure that the cDNAs are labeled with different IL indexes.

***Note:*** Typically, we use 1 μL or 0.2 ng cDNA for each sample.

***Note:*** If the cDNAs have already been mixed after the reverse transcription (see the Alternative note at Step 29), skip this cDNA pooling step.

***Alternatives:*** For full-length sequencing, skip this step (see above and Alternative note at Step 61).

52. Measure the total amount of mixed cDNAs (see Steps 48–50).

***Note:*** If the concentration of the mixed cDNAs is too low to be detected using the Qubit 1× dsDNA HS Assay kit (<0.1 ng/μL), concentrate them using 0.8× volume of AxyPrep MAG PCR clean-up reagent (see Steps 31–41).

53. Dilute the mixed cDNA samples to ensure that their concentration falls within a range of 60–800 pg/μL.
54. Thaw the Tagment DNA Buffer, Amplicon Tagment Mix, and nuclease-free water included in the Nextera XT DNA Library Prep Kit.
55. Prepare the tagmentation mixture in single flat-top 200-μL PCR tubes on ice as follows:

**Table:** Tagmentation mixture

Reagent	Final concentration	Amount
Tagment DNA Buffer	N/A	10 μL
Amplicon Tagment Mix	N/A	5 μL
Nuclease-free water	N/A	5 - X μL
Mixed cDNA (300–400 pg)	N/A	X μL (0.5–5 μL)
**Total**	**N/A**	**20 μL**

56. Place the tubes containing the tagmentation mixture into a thermal cycler and run the tagmentation program.

***Note:*** In this step, TnRP1 and TnRP2 sequences are randomly inserted into cDNAs (Figure 2).

**Table:** Tagmentation program

Steps	Temperature	Time	Cycles
Tagmentation	50°C	5 min	1
Hold	10°C	infinite	1

57. Place the tubes on ice.
58. Add 5 μL of the Neutralize Tagment Buffer to each tube, gently mix it, and briefly centrifuge to collect the contents at the bottom.
59. Incubate the tubes at 25°C for 5 min, and then place them on ice.
60. Thaw reagents for PCR master mix.
  a. Thaw the Nextera PCR Master Mix, as well as the Tn Forward and Reverse PCR primers.
  b. Gently mix the reagents, briefly centrifuge, and place them on ice.
61. Prepare the PCR master mix on ice as follows:

**Table:** PCR master mix

Reagent	Final concentration	Amount
Nextera PCR Master Mix	N/A	15 μL
Reverse PCR primer (P7 index) (10 μM)	N/A	1 μL
Tn Forward PCR primer (P5 index) (10 μM)	N/A	1 μL
Nuclease-free water	N/A	8 μL
**Total**	**N/A**	**25 μL**

***Note:*** As shown in Figure 2, the Tn Forward PCR Primer (P5 index) is specifically designed to hybridize to the TnRP1 sequence, while the Reverse PCR primer (P7 index) hybridizes to the RP2 sequence incorporated into Oligo dT VN IL (Step 12) (see Table S1). This strategy is designed for selective amplification of the 3′-ends of cDNAs in the SMART-seq v4 3′DE Kit.^14^

**CRITICAL:** Ensure that different libraries are labeled with different P5 and P7 indexes.

***Alternatives:*** For full-length sequencing, use the Tn Reverse PCR primer (P7 index) (10 μM) instead of the Reverse PCR primer (P7 index) to amplify all parts of tagmented cDNA (see Table S1). In this case, pooling is not allowed for cDNA in Steps 29 and 51.

62. Add 25 μL of PCR master mix to the neutralized tagmentation product (product of Step 59).
63. Place the tubes into a thermal cycler with a heated lid and run the thermal cycling program for library preparation.

**Table:** Thermal cycling program for library preparation

Steps	Temperature	Time	Cycles
Initial extension	72°C	3 min	1
Initial denaturation	95°C	30 s	1
Denaturation	95°C	10 s	12 cycles
Annealing	55°C	30 s	
Extension	72°C	30 s	
Final extension	72°C	5 min	1
Hold	10°C	infinite	1

**Pause point:** The samples can be stored at −20°C or below for at least 1 week.

64. Purify the amplified DNA samples.
  a. Apply 30 μL of AxyPrep MAG PCR Clean-up (∼0.6× volume) into 1.5-mL sampling tubes at 25°C.
  b. Perform DNA purification following the procedure outlined in Steps 31–39.
  c. Elute the DNA into 12 μL of dH_2_O.

***Note:*** For detailed information, see the manufacturer’s instructions included in the SMART-Seq v4 3′ DE Kit.

**Pause Point:** Samples can be stored at −20°C or below for at least 1 week.

### Part 9. Quality control of the library using an Agilent 2100 Bioanalyzer

**Timing: 1.5 h**

In this step, validate the size distribution and amount of the library DNA. Use of an Agilent 2100 Bioanalyzer and High Sensitivity DNA Kit is recommended.

65. Take 1 μL of the amplified library (product of Step 64) for analysis using the bioanalyzer according to the manufacturer’s instructions.^15^
66. Investigate the size distribution of the library (see expected outcomes).

***Note:*** Other devices for DNA electrophoresis, such as the TapeStation and the Fragment Analyzer systems, can be used in this step.

### Part 10. Quantification of library amounts using real-time PCR

**Timing: 2.5 h**

Quantify the amount of DNA libraries using the KAPA Library quantification Kits according to the manufacturer’s instructions.^16^

67. Prepare the KAPA SYBR FAST qPCR Master Mix with Illumina Primer Mix.
  a. Thaw KAPA SYBR FAST qPCR Master Mix (5 mL) and Illumina Primer Mix (1 mL).
  b. Add 1 mL Illumina Primer Mix to the bottle of KAPA SYBR FAST qPCR Master Mix and thoroughly mix by vortexing.

***Note:*** The mixture can be store at −20°C for at least one year. In our experience, this mixture remains stable through repetitive freeze-thaw cycles.

***Optional:*** Depending on real-time PCR systems, add 200-μL ROX High (for ABI 5700, 7000, 7300, 7700, 7900HT, StepOne, and StepOnePlus) or ROX Low (for ABI 7500, ViiATM 7 and Stratagene Mx3000PR, Mx3005PTM, Mx4000).

68. Quantify the concentration of library DNA samples (products of Step 64) using Qubit 4 Fluorometer with Qubit 1× dsDNA HS Assay kit (see Steps 48–50).
69. Thaw DNA Standards 1–6.
70. Prepare dilution buffer.

**Table:** Dilution buffer

Reagent	Final concentration	Amount
Tris-HCl [pH 8.0] (1 M)	10 mM	0.4 mL
Tween 20	0.05%	20 μL
dH_2_O stock	N/A	39.58 mL
**Total**	**N/A**	**40 mL**

71. Immediately before use, dilute the library DNA samples with the dilution buffer to ensure that their concentrations fall within the dynamic range of the assay provided by the KAPA Library quantification Kits (5.5–0.000055 pg/μL).

***Note:*** We routinely dilute the library DNAs at ratios ranging from 1:2,000 to 1:4,000.

72. Prepare sufficient amount of KAPA premix for library DNA samples and DNA Standards.

**Table:** KAPA Premix

Reagent	Final concentration	Amount
KAPA SYBR FAST qPCR Master Mix with Illumina Primer Mix	N/A	6 μL
dH_2_O stock	N/A	2 μL
**Total**	**N/A**	**8 μL**

73. Apply 2 μL of the diluted library DNA or DNA Standards and 8 μL of the KAPA premix to each well of a 384-well Hard-Shell Microplate (white well/clear shell).

**CRITICAL:** Prepare at least two replicates per sample.

74. Perform real time PCR.
  a. Seal the 384-well microplate with a Microseal ‘B’ sealing film.
  b. Centrifuge the microplate at >1000 × *g* for 1 min to collect the contents at the bottom.
  c. Place the microplate into the CFX Opus 384 Real-Time PCR system.
  d. Run the thermal cycling program to perform KAPA library quantification.

**Table:** Thermal cycling program to perform KAPA library quantification

Steps	Temperature	Time	Cycles
Initial denaturation	95°C	5 min	1
Denaturation	95°C	30 s	35 cycles
Annealing and extension	60°C	45 s

75. Quantify the concentration of library DNA based on the *C*_t_ values obtained from the DNA Standards.

### Part 11. Sequencing analysis

**Timing: 1.5 days**

After quality control and quantification, the library DNAs are ready for sequencing. Libraries labeled with different P5 and P7 indexes can be analyzed simultaneously. In this protocol, we perform 3′-sequencing using NextSeq 500/550 High-Output Kit v2.5 (75 Cycles).

***Alternatives:*** Illumina NextSeq 2000 with P3 Reagents (50 cycles) or any other platforms compatible with the adaptor sequences (e.g., Illumina NovaSeq 6000) can be used in this step.

76. Mix the library DNAs (products of Step 64) labeled with different P5 and P7 indexes at the same concentration, based on the results of library quantification (Steps 67–75).

**CRITICAL:** Ensure that the libraries are labeled with different P5 and P7 indexes.

77. Quantify the DNA concentration using Qubit 4 Fluorometer with Qubit 1× dsDNA HS Assay kit (see Steps 48–50).
78. Apply a 7:3 mixture of the library DNA (product of Step 76) and PhiX Control v3 DNA to NextSeq 500/550 at a DNA concentration of 1.6 pM using the NextSeq 500/550 High-Output Kit v2.5 (75 Cycles).

***Note:*** We routinely perform sequencing with input DNA concentrations ranging from 1.6 to 2.4 pM. For example, we combine 910 μL of 1.6 pM library DNA and 390 μL of 1.6 pM PhiX Control v3 DNA, resulting in a total volume of 1400 μL, which is then applied to NextSeq 500/550.

***Note:*** Incorporating the PhiX Control v3 DNA is recommended due to the low complexity of the index sequences in libraries. Sequencing runs performed without PhiX may lead to incorrect base calling.

79. Perform sequencing in the paired-end mode with parameters set to 68 cycles for read 1, 8 cycles for read 2, and 8 cycles each for indexes 1 and 2.

***Optional:*** If UMIs are used for gene counting, use 18 cycles for read 2.

***Note:*** In our experience, 1–2 million reads per sample provide sufficient coverage for single-cell 3′-end sequencing.

80. Analyze the output sequence data.
  a. Demultiplex the in-line index from the read 2 using SMART-Seq_DE3_Demultiplexer.
  b. Perform quality control of sequence reads using FASTQC^5^ and Fastp.^6^
    ***Optional:*** If UMIs are employed in gene expression analysis, we extract them from the read 2 using umi_tools.^4^ Use dedup option to conduct UMI deduplication.
  c. Generate read counts of genes using htseq-count.^7^
    ***Optional:*** To generate read counts, featureCounts^8^ can be used.

## Expected outcomes

### Staining of ovarian sections (steps 1–9)

Using 1% Cresyl violet/50% isopropanol staining, snap-frozen mouse ovaries yield clear histological images (Figure 5).

### Capturing cells/ROIs from tissue sections (steps 10–18)

If the cells/ROIs have been successfully captured at the cap of the PCR tubes, they can be identified through stereomicroscopic inspection (Figure 7).

### Real-time PCR analysis of cDNAs (steps 42–47)

The typical *C*_t_ values of spike-in RNAs *ERCC00096* fall within the ranges of 17–19 (Figure 8). Robust detection of expression of spike-in RNA indicates successful cDNA amplification.

For oocytes (primary to early antral follicles: 13–100 μm diameter) and granulosa cells isolated from alcohol-fixed ovarian sections, the housekeeping gene *Arbp* shows *C*_t_ values within the ranges of 17–23 and 20–24, respectively. Exceptionally large *C*_t_ values, indicate low expression levels and may suggest a failure in either cell isolation, cell lysis, and/or cDNA amplification.

### Amounts of cDNA and sequencing libraries (steps 48–50)

**Figure 9:**
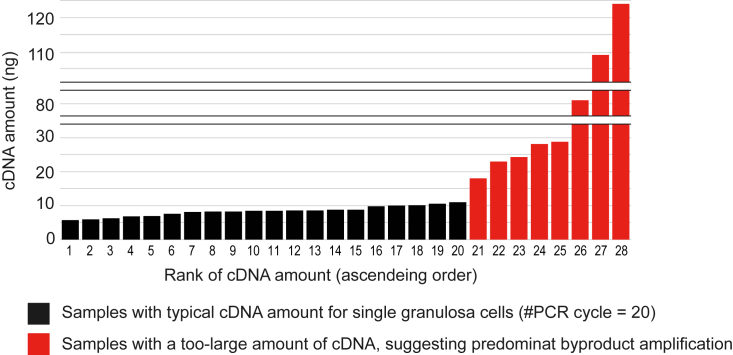
cDNAs amounts of single-cell cDNAs from sections Bar graphs represent amounts of cDNAs amplified from single granulosa cells isolated from ovarian sections (*n* = 28). The cDNA amounts were quantified using Qubit. The purified cDNA samples resulting from the 20-cycle PCR (products of Step 41) are arranged in ascending order for their amounts. The results of samples with too much cDNA due to predominantly byproduct amplification are highlighted in red.

The typical amounts of amplified cDNA samples from single oocytes and granulosa cells after a 20-cycle PCR (product of Step 41) falls within the ranges of 25–50 ng and 5–10 ng, respectively (Figure 9). An excessive amount of cDNA could result from amplification of byproducts, such as primer dimers or concatemers of template-switching oligo nucleotides. cDNA amplification from approximately 300 granulosa cells for 18 cycles would result in a typical amount of 50–100 ng. Additionally, a typical yield of the 3′-end sequencing library from 400 pg of pooled cDNA falls within a range of 20–100 ng.

### Bioanalyzer analyses for cDNA and 3′-end sequencing libraries (steps 65–66)

**Figure 10:**
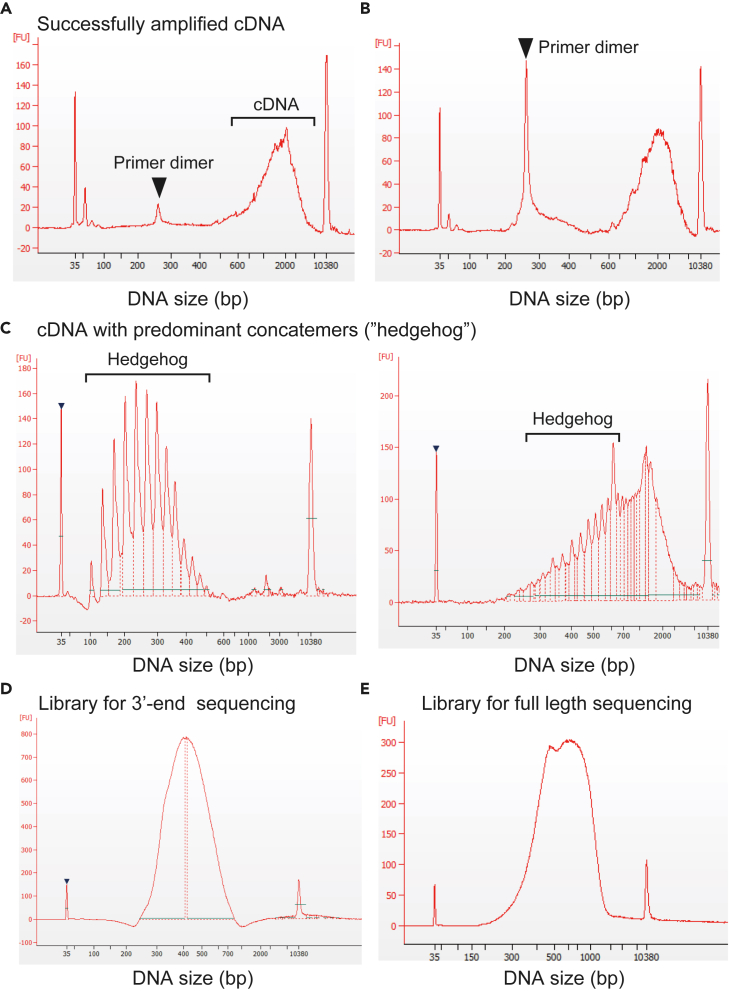
Bioanalyzer electropherogram of cDNA and sequencing library (A) Successfully amplified cDNA. (B) Unsuccessfully amplified cDNA containing a large amount of primer dimer of 200–300 bps. (C) Unsuccessfully amplified cDNA with predominant concatemers (“hedgehog”-type electropherogram). (D) Library for 3′-end sequencing. (E) Library for full-length sequencing.

Typically, the cDNAs (products of Step 41) show electropherograms ranging from 150 to 6,000 bp with a peak at 2,000 bp. The 3′-end sequencing libraries (products of Step 64) exhibit electropherograms spanning from 250 to 800 bp, with a peak at 500 bp (Figure 10). The full-length sequencing libraries have a DNA size of 500–700 bp with a peak of 600 bp.

### Number of detectable genes in 3′-end sequencing (steps 76–80)

**Figure 11:**
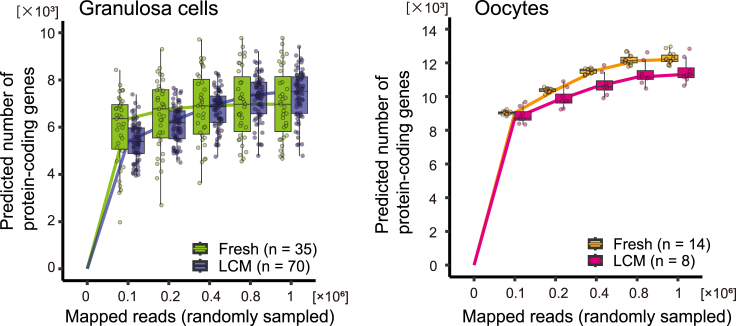
Prediction of detectable protein-coding genes The number of detectable protein-coding genes in granulosa cells (left panel) and oocytes (right panel) isolated from frozen ovarian sections are predicted using rarefaction analysis with random subsampling of mapped reads. The predicted number of genes is plotted for the number of reads.^17^ Extrapolation beyond the range of total mapped reads in each sample was conducted using the module in iNEXT package of R. Sequencing data are available in the NCBI Gene database (GSE192551).^1^ Box and whisker plots are drawn using the default settings of the geom_boxplot function of R; the box spans the interquartile range (IQR) from the first to the third quartile points, while the upper and lower whiskers extend to the maximum and minimum data points within 1.5 × IQR above and below the box, respectively.

The sensitivity of DRaqL-Smart-seq2 from frozen ovarian sections is similar to that of Smart-seq2 from freshly dissociated cells (Figure 11). Typical numbers of detectable genes in granulosa cells and oocytes are >6,000 and >10,000, respectively, when using > 4 × 10^5^ mapped reads.

## Quantification and statistical analysis

### Method for evaluation of successful cDNA amplification

Successful cDNA amplification (product of Step 41) is evaluated through real-time PCR (Steps 42–47, see also expected outcomes and Figure 8). Samples showing exceptionally high *C*_t_ values should be excluded from further steps, as discussed in expected outcomes. To assess these outliers statistically, the Smirnov–Grubbs test can be performed, using the *grubbs.test* function contained in the R program suite.^18^ Samples showing a *p*-value < 0.01 (i.e., a significant outlier) may be considered for exclusion from subsequent analysis.

## Limitations

### Limited throughput of DRaqL-smart-seq2

This protocol offers high-quality RNA-seq for single cells/ROIs isolated from tissue sections. The sensitivity of single-cell transcriptomics from frozen sections with this protocol is comparable to that of freshly dissociated single cells.^1^ However, a few limitations related to this method is relatively low throughput.

The low throughput limitation of this method is due to the cDNA amplification process of Smart-seq2, which is performed using a one-by-one approach in single PCR tubes. Recent single-cell/low-input studies for primate gastrulae, involving approximately 2,000 cells, have been performed with similar efficiency.^19^^,^^20^ Nonetheless, the efficiency could be enhanced by multiplexing the reverse transcription products after Step 29. This is accomplished by leveraging the IL indexes inserted into the reverse transcription primer (Oligo dT VN IL).

Additionally, the method efficiency is constrained by the LCM-based isolation process of cells/ROIs. In the case of granulosa cells in mouse early antral follicles, we need to search for target follicles of interest (typically, 1–2 target follicles per ovarian section meet our criteria: early antral follicles containing oocyte nuclei in the field of view). Consequently, it takes an average of 5 min to find a single granulosa cell in a targeted follicle.

### Possible bias of RNA-seq from tissue sections using DRaqL

There remains a possibility of biases in RNA-seq with DRaqL from fixed tissue sections, which tends to affect a small fraction of lowly expressed genes as quantitatively assessed using alcohol-fixed sections of frozen cell blocks of mESCs.^1^ Except for the low expression levels, we have not identified any distinctive sequence or structural features in the affected genes. Hence, the bias might be attributed to limitations of cell lysis for fixed sections.

### Spatial resolution of single-cell RNA-seq from tissue sections using LCM

The laser cutting edge with an objective lens 63× in this protocol is of approximately 1–2 μm in width. This may limit the spatial resolution for the isolation of cells/ROIs.

## Troubleshooting

### Problem 1

The spike-in RNA could be susceptible to RNase degradation during the dilution processes. Degradation can be detected by real-time PCR after cDNA amplification in Steps 42–47. Low and/or variable expression levels of spike-in RNAs would indicate their degradation and/or failure in amplification, if housekeeping genes are expressed at a high level (Figure 8).

### Potential solution

• To maintain the quality of the ERCC RNA Spike-In Mix solution, aliquot it into a small volume (e.g., 5 μL) in the nuclease-free 1.5-mL sampling tubes shown in the key resources table and store at −80°C.
• To ensure nuclease-free dilution, use dH_2_O from a newly opened bottle.
• Before beginning the dilution, thoroughly chill the dH_2_O on ice for at least 20 min.
• Snap-freeze diluted ERCC RNAs using liquid nitrogen.
• Avoid one-handed tube handling, as it can introduce a risk of RNase contamination into the cap.
• Do not use autoclave or use DEPC-treatment for any materials or equipment for this protocol.

### Problem 2

Tissue sections may roll on the blade, making it challenging to smoothly mount them onto slides (related to Step 3) (Figure 4).

### Potential solution

• Use an anti-roll plate.
• Gently press the sections onto the membrane slide with a soft brush.

### Problem 3

Membrane slides stored at −80°C may experience dew condensation after being returned to 25°C (related to Steps 9, 18) (Figure 6). This could be caused by high humidity.

### Potential solution

• Consider following the Pause-point note at Step 9.
• Use a dehumidifier to maintain a humidity level of 40% or less.

### Problem 4

Tissue sections on the polyethylene naphthalate (PEN) membrane might not be completely cut (related to Step 11) (Figure 5).

### Potential solution

• Ensure the objective lens is correctly focused on the target by microscopic inspection, as optimal focus is crucial for effective laser cutting.
• Consider repeating the laser cutting at a cutting energy level of about 40%. Avoid using excessive energy because it can enlarge the laser width and potentially damage the cellular contents within the section.

### Problem 5

Joints of the section might be accidentally removed (related to Step 11) (Figure 5).

### Potential solution

• Consider reducing the cutting energy to achieve a narrower laser width.
• If use of lower energy results in incomplete membrane cutting, consider repeating the joint cutting.

### Problem 6

Inefficient cDNA amplification, as determined by ERCC RNA quantification at Steps 42–47, may be caused by RNase contamination and/or decreased enzyme activity in the reaction mixtures (cell lysis buffer at Step 12, quenching buffer Step 19, template-switching mixture at Step 20, SeqAmp PCR mixture at Step 27). Thus, it is essential to check for these issues if cDNA amplification is not proceeding efficiently.

### Potential solution

• Ensure that the dH_2_O product is the one specified in the key resources table throughout this protocol.
• The ERCC RNAs are highly sensitive to RNase contamination. Additionally, the protein components (reverse transcriptase, DNA polymerase, and RNase inhibitor) are sensitive to heat denaturation. To protect these components in each reaction mixture from degradation and/or denaturation, first mix all other components, then chill the mixtures on ice for at least 20 min. After that, add protein components to the mixtures immediately before use. In the case of the cell lysis buffer, add the ESCC RNAs after applying the RNase inhibitor.

### Problem 7

Captured cells/ROIs may not fall to the bottom of the tubes during centrifugation (related to Step 16) (Figure 7). This may be due to evaporation of the cell lysis buffer, which can cause the captured cell/ROIs to stick to the tube cap.

### Potential solution

• Perform the LPC and subsequent spinning down of the tubes containing captured cells/ROIs quickly (Steps 13–16). We routinely finish these processes within 1 min.
• Consider increasing the volume of the cell lysis buffer up to 10 μL.

### Problem 8

Inefficient cDNA amplification might result from reduced enzymatic activity due to incomplete quenching of SDc (related to Step 24) (Figure 8).

### Potential solution

• The quenching buffer is highly viscous, so a few taps on the tube may not be sufficient for thorough mixing.
• To guarantee thorough mixing of the quenching buffer and samples, repeat the tapping, and confirm the homogeneity of the suspension by visual inspection before continuing on with the protocol.

### Problem 9

Inefficient reverse transcription may lead to a significant increase in the formation of byproducts, such as primer dimers or concatemers of the template-switching oligonucleotides during cDNA amplification (Step 30). This can be observed in the electropherogram using the bioanalyzer (Figure 10).

### Potential solution

• To reduce byproduct formation, consider reducing the amount of the reverse transcription primer (Oligo dT VN IL) and the template-switching oligonucleotide (N-template-switching oligo), as described in Alternative notes at Steps 12 and 20.
• Another option to examine is the removal of the byproduct DNAs from amplified cDNA (product of Step 30). In this case, repeat the size-selective DNA purification at Steps 31–41 for 2 to 4 times.

## Resource availability

### Lead contact

Kazuki Kurimoto (kurimoto@naramed-u.ac.jp).

### Technical contact

Hiroki Ikeda (ikedahiroki@naramed-u.ac.jp).

### Materials availability

This study did not generate new unique reagents.

### Data and code availability

The accession number for sequence data used in this paper is GEO: GSE192551 as previously reported.^1^

## Supplemental information

Table S1. Oligonucleotide sequences used in this study, related to Steps 12 and 61
